# Therapeutic Approaches of Viral Gene Silencing by Small Interfering RNA: Strategies to Prevent the Emergence of Antiviral Resistant Escape Mutants

**DOI:** 10.3390/ph18070987

**Published:** 2025-07-01

**Authors:** Hara Kang, Yun Ji Ga, Jung Won Kim, Chaeyeon Kim, Se-Hwan Son, Chaeeun Gwak, Jung-Yong Yeh

**Affiliations:** 1 Department of Life Sciences, College of Life Sciences and Bioengineering, Incheon National University, BioComplex, Harmony-ro 265, Yeonsu-gu, Incheon 22014, Republic of Korea; harakang@inu.ac.kr (H.K.); dkfmal92@inu.ac.kr (Y.J.G.); letsgo0429@naver.com (J.W.K.); chaeyeon0719@inu.ac.kr (C.K.); 202202476@inu.ac.kr (S.-H.S.); gce0215@gmail.com (C.G.); 2 Research Institute for New Drug Development, Incheon National University, BioComplex, Harmony-ro 265, Yeonsu-gu, Incheon 22014, Republic of Korea; 3 Convergence Research Center for Insect Vectors, Incheon National University, BioComplex, Harmony-ro 265, Yeonsu-gu, Incheon 22014, Republic of Korea

**Keywords:** antiviral, resistance, RNA interference, small interfering RNA, virus

## Abstract

RNA interference (RNAi) was originally regarded as a mechanism of eukaryotic post-transcriptional gene regulation mediated by small interfering RNA (siRNA)-induced sequence-specific RNA degradation. It is well known to exert as an important antiviral defense mechanism in a wide range of organisms, from plants to invertebrates. The specificity, ease of design, and ability to target conserved gene regions make siRNA technology a promising approach to combat viral pathogenesis, allowing the targeting of multiple virus strains. The mechanism of sequence complementarity utilized by siRNAs against their targets presents a novel strategy to combat viral infections, as they can specifically target and degrade viral RNA. Consequently, siRNA-based therapeutics have been applied to various viral diseases. This is largely due to the design flexibility and rapid response potential of RNAi technologies, which provide advantages over traditional antiviral agents. However, the emergence of viral escape mutants poses a major barrier to the sustained antiviral activity of siRNA-based therapy. Therefore, devising strategies to overcome the emergence of escape mutants to antiviral siRNAs could enhance the efficacy of siRNA-based therapeutics in providing a rapid response to emerging viral infectious diseases. This review aims to comprehensively summarize the current knowledge on siRNA-based therapeutic approaches against viral infections and elucidate the challenges associated with implementing siRNA treatment, with a specific emphasis on antiviral resistance.

## 1. Introduction

RNA interference (RNAi) is a natural mechanism that defends against exogenous gene invasion [1,2], orchestrating the selective silencing of target genes through the degradation of their corresponding mRNA [3]. This process is activated by small double-stranded RNA (dsRNA) molecules, known as small interfering RNAs (siRNAs), which are typically 21–23 nucleotides long. These siRNAs bind to complementary mRNA, thereby inducing mRNA degradation. Introducing exogenous and synthetic siRNAs into cells triggers RNAi, driving RNAi-based therapies against disease-associated mRNA. This spans genetic disorders, acquired diseases, and viral infections [4,5,6,7,8,9,10]. Viral dsRNAs from infection activate RNAi [11]. These dsRNAs yield 21-nucleotide siRNA duplexes, facilitated by Dicer [12]. Within this duplex, one strand, the guide-strand, assembles with the RNA-induced silencing complex (RISC). RISC identifies viral mRNAs, leading to degradation [13,14]. Cleavage occurs around nucleotides 10–11 on the complementary antisense strand [15]. This highly orchestrated pathway underscores the therapeutic potential of siRNA therapy in combating viral infections [1,16].

**Figure 1:**
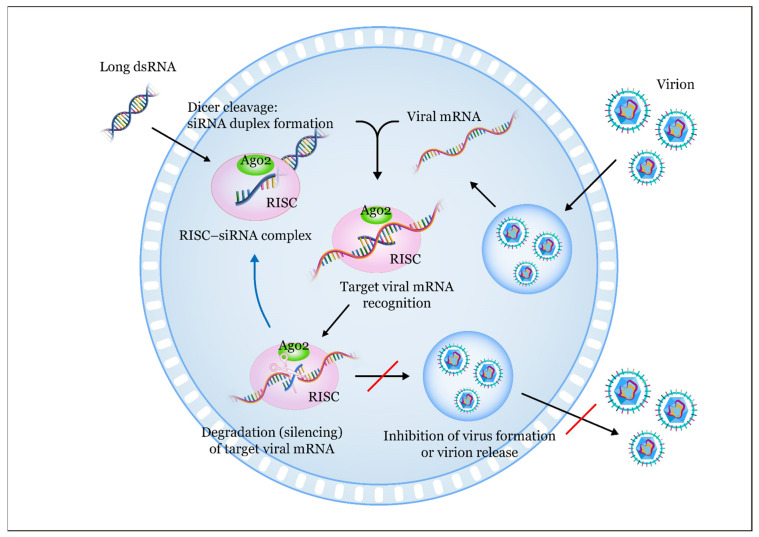
The siRNA-mediated RNA interference (RNAi) pathway against a virus involves several steps: (1) Introduction of antiviral siRNAs: long double-stranded RNAs (dsRNAs) are initially introduced into the cell and subsequently cleaved by the enzyme RNase III (Dicer) in the cytoplasm. This cleavage results in the formation of small dsRNAs; (2) processing into siRNAs: these small dsRNAs are further processed by Dicer, yielding short siRNA molecules that are typically 21–30 nucleotides long [22]. These antiviral siRNAs act as the functional units in the silencing mechanism; (3) formation of siRNA duplexes: Dicer cleavage generates siRNA duplexes, consisting of two complementary strands [23]. These duplexes associate with a multi-protein complex called the RNA-induced silencing complex (RISC) [24,25,26]; (4) RISC assembly: the RNA strands within the siRNA duplexes are separated by the ATP-dependent RNA helicase domain of RISC [27]. One strand, known as the passenger (sense) strand, is degraded within the RISC; (5) guide strand function: the remaining antisense single-stranded RNA, also known as the guide RNA, assists in aligning the RISC with the target viral mRNA. This alignment allows the fragmentation of the RNA sense strand through the action of Argonaute 2 (Ago2); (6) viral mRNA cleavage: the guide strand within the RISC binds to complementary sequences in the target viral mRNA through the Watson–Crick base pairing [25]. Subsequently, the guide strand directs the RISC to cleave the target viral mRNA [28]; (7) impact on gene expression: this cleavage leads to degradation of the target viral mRNA, resulting in the inhibition of viral protein expression [29,30]. Consequently, the expression of targeted viral genes is reduced; and (8) antiviral potential: by targeting viral genes, this mechanism has the potential to inhibit viral replication and infection, making it a promising approach for antiviral therapy [31].

RNAi offers numerous advantages as a therapeutic approach compared to small-molecule drugs, as it holds the potential to target virtually all genes using siRNA molecules. RNAi effectively hinders the replication of a wide range of viruses by using both siRNA and siRNA expression vectors [17]. Due to their ability to exhibit complete base-pairing and induce mRNA cleavage in a precise target region [18], siRNA can effectively inhibit viral infection by targeting specific genes essential for viral entry or replication [19,20]. This results in shorter research and development time and a more cost-effective therapeutic approach. Although targeting the viral genome and its sub-genomic transcripts might not prevent viral entry, this approach still provides protection to uninfected cells by introducing siRNAs that promptly degrade the viral RNA upon its release into the cytoplasm (Figure 1), thereby effectively preventing viral spread [21].

The advantages of siRNA, including its convenient design and production, make it a promising alternative to existing vaccines and antiviral compounds for targeting infectious diseases [32,33]. This readily adaptable approach is particularly beneficial for targeting viruses with rapidly varying dominant genotypes [34] and phenotypes. It is particularly valuable in situations where predicting vaccine efficacy is difficult, or in seasons when available vaccines inadequately match the circulating viral strains. siRNA-based therapies could potentially serve as a first line of defense against viral strains that acquire resistance to previously effective drugs and vaccines, either through natural evolution or human intervention [17]. This review is specifically focused on strategies to overcome the emergence of escape mutant viruses in the context of siRNA-based antiviral therapy. By narrowing the focus to resistance-related mechanisms, this review aims to provide a more in-depth and actionable framework for overcoming one of the most pressing barriers to siRNA-based antiviral therapies.

## 2. Challenges of Emergence of Resistant Escape Mutants to Antiviral siRNAs

siRNA therapeutics have emerged as a promising therapeutic class for antiviral treatment, offering target specificity and lower side effects, especially in the context of emerging infectious diseases [35]. However, the emergence of viral escape mutants poses a significant obstacle to the therapeutic efficacy of siRNA-based antiviral therapy.

### 2.1. Emergence of Escape Variants to Antiviral siRNAs

A challenge for the development of siRNA therapeutics with antiviral activity is that viruses mutate rather quickly, which frequently allows them to alter the target sequence of the siRNAs and severely reduce or completely abolish the efficacy of RNAi [36,37,38,39]. This can occur through mutations in the target viral gene and render siRNA-based antiviral treatments ineffective towards the new variants. Viral escape variants are viral strains that have developed mutations in the sequence targeted by siRNA, making the siRNA unable to effectively suppress viral replication. Especially, RNA viruses encode polymerase enzymes, such as an error-prone RNA-dependent RNA polymerase, that lack proofreading abilities and as a result have high rates of mutation. Consequently, RNA viruses accumulate point mutations up to 10^7^-fold more rapidly compared to viruses with DNA genomes [40]. Thus, there is a high probability that viruses with resistance to RNAi induced by a particular siRNA will evolve during the course of virus replication by nucleotide mutations in the target sequence of the siRNA.

### 2.2. Selection of Escape Mutant Viruses Due to Prolonged RNAi Activity

Evidence from several studies indicates that the antiviral activity of some siRNAs may be more limited than initially predicted. Westerhout et al. showed that human immunodeficiency virus-1 (HIV-1) escape variants emerged after prolonged culturing at a higher level of resistance than expected, and that these mutations induce alternative folding of the RNA so that the target sequence is occluded from binding to the siRNA, resulting in reduced RNAi efficiency [38,41]. These resistant viruses contained nucleotide substitutions or deletions in the target gene, modifying or deleting the siRNA-target sequence. Additionally, an inverse correlation between the level of resistance and the stability of the siRNA/target-RNA duplex was observed in siRNA-resistant escape variants [41]. Xue and colleagues demonstrated that the precise targeting of siRNAs to specific sequences, along with prolonged treatment, can facilitate the emergence of siRNA-resistant viral variants, especially in viruses that encode a polymerase which lacks proofreading capabilities [42]. Studies have reported the selection of escape mutant viruses in response to prolonged RNAi activity [38,39] as well as instances of single treatments [37]. These findings highlight the enormous genetic flexibility of viruses and provide detailed molecular insight into the sequence specificity of RNAi and the impact of target RNA secondary structure.

### 2.3. Reduction in RNAi Efficiency by Mutation of the siRNA Target Sequence

Mutations in the RNA sequence can lead to changes that prevent the binding of siRNA, making it resistant to siRNA-mediated degradation. The major mechanism of viral resistance to siRNA therapeutics occurs through mutations in the target viral gene. Escape variants that are resistant to siRNA therapy typically contain point mutations, diverse deletions, or nucleotide substitutions in or near the targeted sequence [38,41,43]. The efficacy of RNAi interaction can be diminished by mutations in the target sequence that cause mismatches with the siRNA or induce changes in RNA structure in which the target sequence is occluded. Mutations in viral target RNA sequences influence the efficacy of RNAi to varying degrees, depending on the mismatch position within the siRNA–target duplex [44,45]. Kinetic analyses have shown that different regions of the siRNA have specific functions in terms of target recognition, cleavage, and product release [46]. The initial binding of the siRNA to the target RNA is influenced by the 5′ end, while mutations in either the central or the 3′ region of the target have the most significant impact on the efficiency of RNAi [44,45].

The spike protein of severe acute respiratory syndrome coronavirus-2 (SARS-CoV-2) has been considered as a promising target for RNAi-based inhibition due to its fundamental role in cell infection [47,48,49,50]. The spike protein of SARS-CoV-2 is under high selection pressure, leading to a high number of mutations [51,52,53,54]. This protein is prone to mutations, increasing virus fitness and contributing to the emergence of different variants of SARS-CoV-2 (e.g., alpha (B.1.1.7), beta (B.1.351), gamma (P.1), and delta (B.1.617.2) [55]. These findings revealed that viruses can evade siRNA-mediated inhibition not only through nucleotide substitutions or deletions in the siRNA target sequence, but also through mutations that modify the local secondary structure of the RNA.

### 2.4. Ecological and Evolutionary Drivers of Viral Escape from RNAi

siRNA-based antiviral strategies may inadvertently contribute to the emergence of viral escape mutations, particularly in genetically diverse host populations. For instance, in the turnip mosaic virus, escape mutations were observed in resistant hosts, especially when they coexisted with susceptible ones, highlighting the role of ecological context and mixed host populations in driving viral evolution [56]. Additionally, hosts with varying expression levels of RNAi-mediated artificial miRNAs exhibited differential susceptibility, further demonstrating that host genetic diversity can influence viral adaptation.

Viral hosts naturally form genetically diverse populations through evolution and mutation, exerting continuous evolutionary pressure on viruses. For example, in natural populations of *Arabidopsis thaliana*, the argonaute 2 gene—an essential component of the RNAi pathway—exhibits high levels of polymorphism. These genetic variations are associated with differences in susceptibility and resistance to the potato virus X (PVX) [57,58]. Such findings underscore the role of host–virus co-evolutionary dynamics in shaping viral evasion of RNAi.

Moreover, viruses can adapt to RNAi-based defenses through both short-term mutations and long-term evolutionary mechanisms. Holz et al. demonstrated that resistance to siRNA treatment emerged within just 3–10 passages via single or double nucleotide substitutions [59]. Similarly, Aguado et al. showed that RNA viruses can evade RNAi rapidly through homologous recombination [60], indicating that viral genomes possess intrinsic mechanisms for adaptation. These findings collectively suggest that RNAi evasion can occur via both immediate genetic changes and sustained evolutionary responses to ecological pressures.

Taken together, these insights highlight that RNAi-based therapeutics may face resistance not only due to short-term mutational events but also as a result of broader ecological dynamics and long-term viral evolution in natural environments.

## 3. Strategies to Prevent Emergence of Escape Variants to Antiviral siRNAs

In the face of the ever-changing viral mutations, siRNA therapeutics remain an attractive option for antiviral treatments due to their target specificity and reduced side effects [35]. The emergence of viral escape mutants poses a significant challenge to the efficacy of siRNA-based antiviral therapy, highlighting the importance of carefully selecting siRNA target sequences during treatment strategy development. To address this challenge and respond rapidly to emerging infectious diseases like COVID-19 [61], it becomes crucial to develop well-targeted and highly effective therapeutics by meticulously selecting siRNA target sequences (Table 1). Several strategies have been proposed to prevent or delay the emergence of viral escape mutants, as follows.

### 3.1. Strategies to Prevent or Delay the Emergence of Escape Variants to Antiviral siRNAs

#### 3.1.1. Using Multiple Antiviral siRNAs

Similarly to current antiviral drug combination therapies for viruses with high genomic variability, using multiplex siRNAs can impede the development of escape mutants that are resistant to RNAi-mediated inhibition [34,129,130]. Ideally, employing multiple siRNAs that target essential viral sequences should be considered to prevent or delay the emergence of escape variants to antiviral siRNAs. Using multiple siRNA targets in combination can improve antiviral efficacy compared to single-target approaches. This enhancement occurs through various mechanisms, including broader coverage of viral variants and reduced likelihood of resistance [37,130,131,132].

In contrast to small molecule-based approaches, the siRNA-based approach allows targeting of multiple viral sites at once, reducing the risk of escape variants. Multiplexing siRNA targets in a siRNA combination therapeutic could increase protection by providing redundancy, which means that if one target does mutate, the other target will still provide protection [133,134]. The benefits of employing multiple siRNA antiviral approaches over single treatments are as follows [17]:

(1) Synergistic Inhibition: Targeting multiple loci within the same gene has been shown to produce a synergistic suppression of viral replication. [62].
(2) Enhanced Inhibition: By simultaneously targeting several viral genes and blocking multiple steps in the viral life cycle, enhanced inhibition can be achieved [63,64].
(3) Reduced Emergence of Resistant Mutants: The accumulation of multiple mutations would be required for the virus to overcome this potent inhibition, significantly reducing the likelihood of a fully resistant mutant emerging [37,76,77].

For example, the multiple siRNAs combination treatment approach has also been utilized for HIV-1 to address the highly diverse virus sequences and subtypes globally [133,135]. Several studies also revealed that siRNA targeting multiple viral genes could efficiently inhibit viral replication, and the antiviral effect was markedly stronger when interfering with multiple viral genes [34,71]. Wilson et al. demonstrated that using a combination of two siRNAs severely limited the evolution of escape mutants, suggesting that siRNA activity could be used as a treatment to mitigate the devastating effects of viral infection on affected organ tissue, and multiple siRNAs could prevent the emergence of resistant viruses [37].

Recent studies have shown that siRNAs targeting multiple viral sites at once can effectively suppress rapidly evolving viral RNAs, such as SARS-CoV-2 [65,66,67]. For example, Wu et al. designed 11 siRNAs targeting consensus regions of three key SARS-CoV-2 genes: spike, nucleocapsid, and membrane. Most of the siRNAs significantly decreased the expression of viral genes within 24 h, with inhibition rates exceeding 50% [67]. Idris et al. screened multiple siRNAs, targeting highly conserved regions of the SARS-CoV-2 virus, and identified three candidate siRNAs against viral helicase, RdRp, and the 5′ untranslated region (UTR) that effectively inhibited the virus by over 90%, either alone or in combination with one another [72]. He et al. reported that targeting multiple structural genes with multiple siRNAs, rather than using single siRNA at the same total dosage, synergized antiviral effects against severe acute respiratory syndrome coronavirus (SARS-CoV) [73,74].

Xing and colleagues demonstrated that the combination treatment with dual siRNAs against different regions of the hepatitis C virus (HCV) gene achieved synergistic inhibition effects on viral replication at low doses, compared to single siRNA treatment [75]. The authors mentioned that HCV can escape siRNA silencing through the accumulation of nucleotide point mutations within the siRNA target sequence, rendering siRNA targeting of a single viral gene region ineffective after long-term treatment with the same siRNA. The combinatorial use of multiple siRNAs has been shown to not only exert synergistic suppression of HCV replication but also address issues of antiviral resistance.

#### 3.1.2. Using siRNA Targeting Highly Conserved Regions

Several factors need to be considered to enhance the therapeutic efficacy of siRNA treatments. Firstly, their binding targets should be highly conserved to decrease the possibility of viral escape caused by viral evolution [24], because most viruses possess genomic regions that are less likely to tolerate mutations despite the high mutation rate. By employing siRNA that targets functionally essential and highly conserved regions, this issue can be further mitigated, reducing the number of siRNAs required and consequently lowering the cost [89].

Designing specific siRNAs for highly conserved viral genome regions can resist viral infections with high mutation rates, which is considered one of the advantages of RNAi therapy [136]. Therefore, when potentially targeting conserved viral domains, the fast mutation rate of the viral genome must be considered with the help of available libraries and computational resources [61,79,80]. Using a bioinformatics approach to predict potential mutations is common to design siRNAs that target highly conserved regions of the viral genome.

siRNA is capable of inhibiting a wide spectrum of viral variants, making it a potential one-for-all therapy for rapidly evolving viruses [90]. Numerous studies have revealed that siRNAs targeting highly conserved regions of viral genomes can efficiently inhibit the replication of various viruses, including the chikungunya virus [137,138], coxsackievirus [139], dengue virus (DENV) [84], HCV [81,105], hepatitis B virus (HBV) [89], human enterovirus B [140], HIV-1 [92], influenza virus [141], Japanese encephalitis virus [85], Lassa virus [142], respiratory syncytial virus [143], and the West Nile virus [144,145,146,147,148]. In addition, siRNA therapeutics targeting the highly conserved region of SARS-CoV-2 have been suggested to be effective [78]. Thi et al. demonstrated that a single siRNA can protect against multiple sequence-divergent variants of the Marburg virus and Ravn virus, representing a proof of principle for the utility of rational design in identifying siRNAs with broad-spectrum activity, a key milestone in progress toward RNAi therapeutics [149,150].

In some cases, “pan-viral” siRNAs can potentially inhibit the replication of multiple viruses, including emerging viruses for which limited information is available. For instance, the RNA-dependent RNA polymerase of SARS-CoV has a 96% similarity to that of SARS-CoV-2, an important finding that can be exploited as a target for siRNA [91]. The similarity between the genomes and the highly conserved sequences allows RNAi studies on SARS-CoV to provide key insights into the design of siRNA-based SARS-CoV-2 therapy [33]. Because the secondary structure of the 5′UTR of SARS-CoV and SARS-CoV-2 is remarkably similar, it can be the most important target for RNA-based therapeutics [86]. Similarly, the cis-acting replication element sequence within the 2C-coding region of human enterovirus B has been considered as a highly conserved region targeted for RNAi to inhibit viral replication [140]. Lee et al. suggested that siRNAs, specific to the cis-acting replication element sequence within the 2C-coding region of human enterovirus B, can be a powerful pan-enteroviral treatment [140]. Furthermore, Leonard et al. described that because viruses often contain complex gene regulation circuits that exhibit nonlinear behavior [151], targeting specific loci in this circuitry may optimally suppress viral replication [17].

Targeting a strongly conserved region, such as the untranslated region crucial for viral RNA replication and transcription, was considered a promising approach. Several studies have reported the importance of both the 5′ UTR and the 3′ UTR for viral RNA transcription and replication. For example, highly conserved RNA sequences that should be constrained for viral escape exist in the 5′ and 3′ non-translated regions of HCV. Randall et al. described that multiple siRNAs, targeting these evolutionarily conserved RNA sequences, may limit the emergence of escape mutants [81,82,83].

The 5′ UTR, containing a leader sequence of a virus genome, can become an effective target for the development of siRNA-specific therapies against viral infectious diseases [36,87]. By targeting highly conserved regions, such as the 5′ UTR, the likelihood of the emergence of viral escape mutants can be reduced since these regions are less likely to accumulate mutations that confer resistance to siRNA-mediated targeting [85]. The siRNA antiviral therapeutics could also target the leader sequence of viral genome, inhibiting the viral replication [4]. In this context, Stein et al. found that a synthetic siRNA targeting the highly conserved 5′ cyclization sequence region of DENV genome reduced the viral replication of its four serotypes [84]. It was also reported that the siRNA targeting the leader sequence of SARS-CoV-2 was more effective than the siRNA they developed in parallel targeting the spike protein gene [32]. Some studies have also reported the importance of the 3′ UTR in addition to the 5′ UTR for viral RNA transcription and replication [33,86]. siRNA therapeutics targeting the spike protein of SARS-CoV was the most efficient way to inhibit the replication; however, a research group revealed that siRNAs targeted against the 3′ UTR could also suppress SARS-CoV replication [88].

### 3.2. Strategies for Rapid Adaptation of siRNAs in Response to Target Viral RNA Sequence Mutations

#### 3.2.1. Antiviral Resistance Screening

Viruses undergo rapid replication and often accumulate mutations and small deletions, particularly those with RNA genomes in their life cycles. The efficacy of siRNA in suppressing viral activity relies on a close match between the siRNA guide and the target mRNA. However, the accumulation of mutations can lead to viral resistance against RNAi suppression. Screening for resistance to siRNA therapy can identify the emergence of escape variants and enable the development of new siRNA sequences that are effective against multiple viral strains, including those that have acquired mutations. Viruses can acquire resistance through silent point mutations, altering the nucleotide sequence even without impacting the encoded amino acid. These silent mutations have the potential to disrupt the recognition of the target RNA by the siRNA. Gitlin et al. demonstrated that escape viruses (resistant to siRNAs) can emerge from a single nucleotide substitution within the targeted sequence [43].

The efficiency of RNAi can be affected by the position of mismatched bases resulting from silent mutations and their nucleotide identity. Du et al. showed that G:U wobble base pairing is generally well tolerated for RNAi at most positions [93]. Conversely, Gitlin et al. reported that an A→G mutation, which produced a G:U mismatch, eliminated RNAi more efficiently than an A→C or A→U substitution [77]. Additionally, von Eije et al. observed the preferential selection of mutations that cause a relatively weak disruption of the target-siRNA duplex with G-U base pairs and C-A mismatches [92]. The adjustment of the siRNA sequence allows a rapid adaptation of their antiviral activity against different variants of concern. The ability to attach siRNAs to receptor ligands through click chemistry was shown to facilitate the construction of targeted siRNAs, enhancing the flexibility of antiviral defense strategies [65].

#### 3.2.2. Identification of New Targets

In response to viral resistance, another strategy involves swiftly identifying new targets for siRNA therapy. For this challenge, siRNAs have the advantage that they can be rapidly adjusted if mutations occur in the viral target RNA sequences [78,152]. The development of siRNA therapeutics can begin promptly once the genome sequences of escape variants are identified, while the development of small molecule therapies that target complex protein activities necessitates a more comprehensive understanding of the biological and functional details of mutant viruses. This advantage makes siRNA-based therapeutics a promising concept to provide many new targets against constantly evolving viruses.

Wilson et al. demonstrated that variants that resisted one siRNA after repeated treatments remained fully susceptible to another siRNA directed against a different target sequence [37]. They also described that the target RNA did not succumb to RNAi activity immediately and replicated in the presence of siRNA to subsequently develop escape mutations. O’Brien’s findings suggested that escape mutants are not easily generated when specific conserved regions of the sequence are targeted, as mutations were not observed in the siRNA target sites [94]. On the other hand, Westerhout et al. described an instance where an HIV mutant escaped RNAi suppression by accumulating mutations outside the target sequence, leading to the creation of a new local RNA secondary structure [41]. It is of course possible that viruses may have an inherent resistance to RNAi at any time.

The accurate and reliable estimation of the mutation rate of viruses is essential to understand their evolution and to develop siRNA strategies to combat them. This variability and rapid evolution can allow viruses to acquire antiviral resistances, posing significant challenges in designing effective siRNA therapeutics against diseases. Hence, identifying new targets must consider the fast mutation rate of the viral genome, potentially targeting conserved viral domains. Moreover, screening a library of siRNA sequences against a panel of viral strains, encompassing both wild-type and mutated strains, to pinpoint siRNAs that can effectively target multiple viral strains is advisable [61].

Recently, Zhang et al. proposed a model in which pre-designing siRNAs could enable testing for efficacy and safety prior to future outbreaks of newly emerged viruses [95]. This kind of model can expect that the more information is available, the better the pre-design concept can work and, thus, will be very useful in identification of new viral regions targeted by siRNAs. It will also be helpful in saving money and time in clinical trials, thus accelerating responses toward future emerging viruses.

#### 3.2.3. In Silico (Computational) Design for Optimal siRNA Candidates

A library of siRNA sequences is systematically screened against a panel of viral strains, encompassing both wild-type and mutated strains. This screening aims to identify siRNAs with the capability to efficiently target a range of viral strains. The efficacy of siRNAs against a panel of viral strains can be evaluated by performing in vitro screens using cell-based assays and in vivo screens, such as animal models. An approach that is direct, but inherently expensive, entails conducting batteries of in vitro assays to assess the susceptibility of viral isolates.

A more cost-effective approach involves employing statistical bioinformatics analysis of the viral genotype, specifically focusing on the dominant viral genetic sequences present in isolates [107]. Bioinformatics has significantly contributed to designing efficient siRNA sequences for various pathogenic viruses. Diverse computational methodologies rooted in bioinformatics, such as stochastic models [76,96], viral siRNA databases [97,98], siRNA analysis tools [98,99], prediction algorithms [153], and web-based software for antiviral siRNA design [100], have been developed to craft optimal antiviral siRNAs.

The utilization of in silico siRNA design allows for the prediction of highly effective siRNAs prior to their synthesis and subsequent biological testing. Recent advancements have bolstered the predictive capacity of these bioinformatics methods by explicitly considering viral evolution. These approaches usually rely on extensive datasets to establish a connection between viral genotypes and the previous outcomes of specific siRNA candidates [106]. This correlation helps in selecting the most effective siRNAs for optimal targeting. In silico (computational) studies for optimal design, followed by selection with experimental validation, have been performed to further improve the siRNA design [105,154].

ElHefnawi and colleagues devised an enhanced computational screening process to design effective and specific siRNAs targeting the highly conserved regions of viral genomes [105]. The researchers demonstrated that their in silico design and selection protocol could take into account various factors, including variations in target RNA, thermodynamics, accessibility of the siRNA and target RNA, and potential off-target effects. The procedure involved designing and filtering siRNAs that specifically targeted regions of the viral genome, such as the internal ribosome entry site and adjacent core sequences, which were highly conserved and accessible. The selected siRNAs were assigned high-ranking efficacy scores based on their performance.

Recently, a data-based computational algorithm supporting the pre-design of anti-viral siRNA therapeutics before viral outbreaks has been developed. Zhang et al. proposed the creation of a repertoire of pre-designed, safety-tested, and ready-to-use siRNAs that could accelerate responses toward future viral diseases [95]. The authors outlined a computational pipeline facilitating the rational design of siRNAs targeting current and prospective respiratory viruses. The algorithm factors in considerations of siRNA properties, off-target effects, viral RNA structure, and viral mutations.

Yogev et al. embarked on a screening endeavor involving over 16,000 RNAi triggers aimed at the genomes of SARS-CoV-2, HIV, and HCV, with the goal of pinpointing hyper-potent candidates [101,102,103]. This large-scale screening employed a massively parallel assay, using a synthetic biology system to simulate the silencing activity of each siRNA candidate against the virus. The authors described that extensive computational analyses and in vitro experiments yielded a cocktail of hyper-potent siRNA candidates, effective against all tested viral strains, proving that proving to be active against different strains of the virus [101]. El-Kafrawy and colleagues demonstrated that predicting siRNAs against the Middle East respiratory syndrome coronavirus (MERS-CoV) using online software for rational design accurately selects potent siRNAs [104]. They described how the use of online software for rational design aids in the filtration and selection of potential siRNAs with high accuracy and strength.

### 3.3. Strategies to Enhance the Efficacy of siRNA-Based Antiviral Therapeutics

The efficacy of siRNA-based antiviral therapeutics stands as one of the important factors in the development of antiviral-resistant escape variants. When the efficacy of siRNA-based antiviral therapeutics is not appropriate or lower than expected at a given concentration, it also cannot block RNAi activity completely and it, thus, can increase the likelihood of developing escape variants resistant to the antiviral siRNAs. This is because sub-optimal efficacy of the siRNAs can promote the selection of viruses that are able to tolerate or resist the siRNAs. In other words, it is important to avoid using siRNAs with lower efficacy because it increases the risk of triggering viral resistance. Konishi et al. postulated that viral resistance to antiviral siRNAs could result from the low efficiency of siRNA delivery or limited accessibility of the target genome by the siRNA molecule [10].

**Figure 2:**
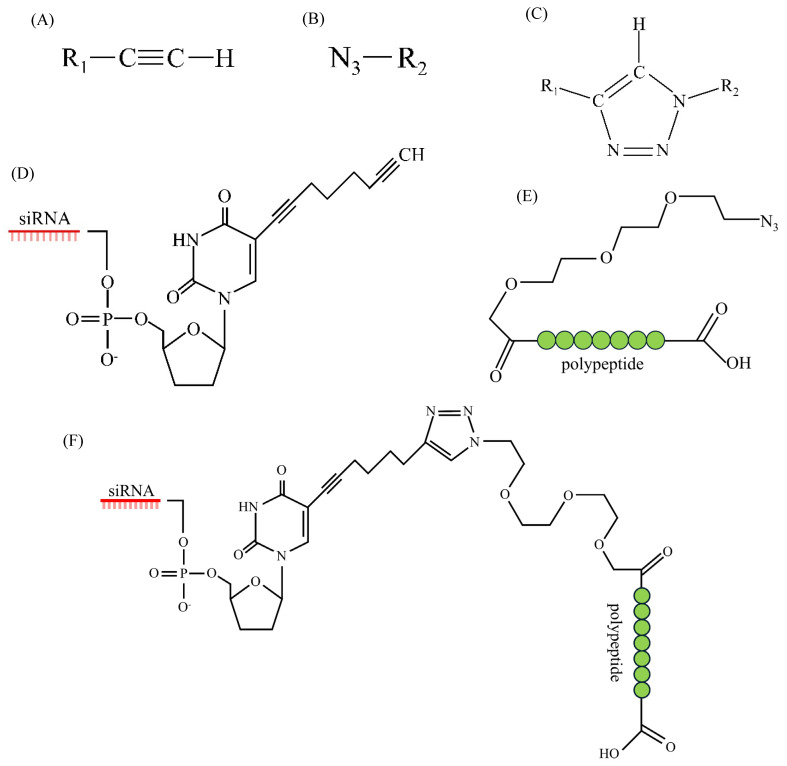
Schematic representation of siRNA–polypeptide conjugation using copper(I)-catalyzed click chemistry. (**A**) Alkyne functional group; (**B**) azide functional group; (**C**) regioselective triazole formation via Cu(I)-catalyzed azide–alkyne cycloaddition; (**D**) siRNA molecule functionalized with an alkyne group; (**E**) polypeptide functionalized with an azide group; (**F**) covalent conjugation of siRNA and polypeptide through click chemistry.

Given the susceptibility of RNA molecules to degradation by host nucleases, improving RNA stability is a key concern in the development of siRNA therapeutics. Various strategies have been developed to overcome this limitation (Figure 2). Chemical modifications such as 2′-O-methyl and 2′-fluoro modifications, as well as phosphorothioate linkages, improve nuclease resistance and increase binding affinity to target RNA. Encapsulation in lipid-based nanoparticles can protect siRNA from serum nucleases and enhance cellular uptake. Furthermore, advanced conjugation strategies and rational sequence design minimize recognition by innate immune sensors. These approaches collectively contribute to the improved stability and efficacy of siRNA-based antiviral treatments [155].

#### 3.3.1. Combination Therapy

RNAi operates in a dose-dependent manner, but even at high concentrations, siRNAs are often insufficient to completely suppress the expression of a target gene [156]. Consequently, their ability to inhibit viral replication is inherently limited [108]. To enhance the efficacy of RNAi-based antiviral therapeutics, siRNAs can be combined with other antiviral approaches that target critical steps in the viral life cycle, as proposed in [68,69,70]. A combination of siRNA-based therapy with other antiviral agents, such as small molecule inhibitors or neutralizing antibodies, can be used to reduce the emergence of viral variants that have acquired mutations that render them resistant to siRNA-mediated targeting. This combination has the potential to significantly increase therapeutic efficiency. Alternatively, it could enable the use of lower doses of antiviral drugs, thereby reducing the risk of drug-induced side effects.

For instance, He et al. reported that the combination of IFN-a with siRNAs against either the replicase or membrane 1 protein reduced titers of SARS-associated coronavirus in infected cell lines by 100 to 1000 times more than siRNA alone [74]. Pozzuto et al. highlighted that the efficacy of siRNA-based RNAi therapy can be further enhanced by combining it with a virus receptor trap and the antiviral drug cidofovir [108]. This underlines the potential of synergistic effects when combining siRNA-based therapy with other antiviral agents to increase the efficiency of antiviral siRNAs. In a recent study, Yuen and colleagues demonstrated that a combination therapy involving siRNAs targeting HBV RNAs along with nucleos(t)ide analogs was well tolerated and led to significant reductions in HBV surface antigen levels in enrolled patients [109].

#### 3.3.2. Modified siRNA Design

Modifications to the siRNA design can enhance its stability, cellular uptake, and target specificity [24,33,110,111], which can increase the efficacy of the treatment and concurrently reduce the risk of viral escape variants as well as its toxicity and immunogenicity [3]. Chemical modifications to the siRNA backbone or the introduction of conjugates can improve the pharmacokinetic properties and cellular uptake of siRNAs. Conjugates hold the capability to facilitate selective siRNA delivery into the target tissue, to adapt to the cellular environment and to avoid endosomal sequestration [113]. A diverse range of bioconjugates have been employed for in vitro and in vivo siRNA delivery, encompassing nanoparticles, aptamers, peptides, cholesterol, polyethylene glycol, and immune proteins [114].

A novel technique devised by Zhou and his colleagues involved the addition of a ‘sticky’ sequence to a chemically synthesized aptamer, enabling the attachment of Dicer substrate siRNAs to allow potential multiplexing [112]. The authors explained that this bridge-base approach provides a significant advantage in terms of chemical synthesis and the ability to bind the aptamer with various siRNAs in a non-covalent manner. They further noted that this approach has the potential to enable the combination of different siRNAs with a single aptamer, allowing for multiplex targeting to prevent resistance to the siRNA component.

siRNAs can be chemically engineered using Cu(I)-catalyzed click chemistry to attach functional moieties, such as receptor-binding peptides, thereby enhancing their stability, cellular uptake, and targeting efficiency [157,158,159]. For example, Traube et al. demonstrated that peptide-conjugated siRNAs generated via this approach retained gene-silencing activity and significantly suppressed SARS-CoV-2 replication in vitro, reducing viral load and virus-induced cytotoxicity by up to five orders of magnitude [65]. These findings underscore the versatility of siRNA platforms and their potential to be rapidly adapted for emerging viral variants.

Moreover, advancements in sequence specificity using cutting-edge technologies like unlocked nucleic acid modification or modulating seed-pairing through thermally destabilizing chemical modification can considerably enhance RISC’s recognition of siRNA [115], thereby mitigating off-target effects [116]. Khaitov et al. showed that the incorporation of locked nucleic acid modifications enhances the RNA stability, and complexation with the designed peptide dendrimer, enhances cellular uptake to allow topical application by inhalation of the final formulation. The authors demonstrated a significant reduction in virus titer and lung inflammation in animals exposed to inhalation of the siRNA [117].

The therapeutic application of siRNA is limited by several obstacles. This is primarily because siRNA, being negatively charged, is impermeable to cellular membranes, highly unstable in systemic circulation, and susceptible to enzymatic degradation by serum endonucleases and RNases [160]. Therefore, extensive efforts have been devoted to the chemical modification of siRNAs and the optimization of delivery vehicles [155].

The naturally occurring 2′-O-methyl (2′-O-Me) modification of RNA enhances binding affinity and resistance to nucleases [161], while also diminishing immune stimulation [111]. With 2′-O-Me as the foundation, medicinal chemists investigated alternative 2′-O-alkyl substituents, identifying 2′-methoxyethyl (MOE) and 2′-fluoro (2′-F) modification as effective analogs [162]. These modifications contributed to a further increase in nuclease resistance and elevated the melting temperature on a per-base basis, thereby enhancing binding affinity to the target RNA [163]. Consequently, the majority of therapeutic siRNAs currently undergoing clinical trials incorporate 2′-MOE or 2′-F modifications [1].

Regardless of chemical modifications, the size and hydrophilic nature of siRNA molecules create significant barriers to systemic circulation [164]. To overcome these issues and efficiently deliver siRNA to target cells, appropriate delivery agents are required [165]. Therefore, siRNAs have been delivered by encapsulation in synthetic carriers, such as cationic liposomes or nanoparticles, or by conjugation with cell-penetrating peptides or antibodies specific to the infected cells [166]. Polymer-based siRNA delivery vehicles provide considerable flexibility and protect siRNAs from nuclease degradation. Likewise, inorganic nanoparticles are recognized for their small size and enhanced permeability [167].

Recently, stable nucleic acid lipid particles have emerged as effective tools for the stabilization and efficient delivery of siRNAs [97]. A comprehensive understanding of the chemical strategies underlying the development of siRNA-based drugs is essential for informing future clinical candidate design and ensuring the sustained success of siRNA therapeutics [168].

#### 3.3.3. Enhanced Delivery Strategies

The potential of using antiviral siRNA for therapeutic purposes is promising, given its ability for precise and effective gene silencing. However, several intracellular and extracellular challenges must be overcome. A significant obstacle in utilizing siRNA as a therapeutic agent involves developing appropriate delivery methods. A successful delivery strategy for siRNAs needs to address various limitations, including concerns about stability and the stimulation of undesirable innate immune responses [169]. The therapeutic efficacy of siRNA-based antivirals relies heavily on efficient intracellular delivery, which remains a key challenge in reaching infected cells and tissues.

Nanoparticles are used as carriers to deliver functional nucleic acid fragments into targeted sites within the body [114,118], thus enabling precise and focused therapeutic interventions [124,125,126,127]. The nanocarrier systems can be formed as vesicle-based nanoparticle suspensions post-encapsulation, thereby improving the stability of the small-molecule drugs contained therein [128]. Combining the targeting properties of nanoparticles with the specificity of RNAi could allow for the design of products with better therapeutic efficacy with lower side effect profiles [114]. Delivery challenges can be mitigated by delivering siRNAs using organic origin nanocarrier systems such as lipid-based nanoparticles [72,119,120,121]. Moreover, cutting-edge nanocarriers have integrated inorganic nanoparticles, encompassing magnetic nanoparticles [170,171], gold nanoparticles, carbon-based nanoparticles, ceramic nanoparticles, metal nanoparticles [172,173,174], and semiconductor nanoparticles [114,122,123,175,176].

## 4. Concluding Remarks and Future Perspectives

Emerging infectious diseases pose a serious threat to public health and pandemic preparedness. The COVID-19 pandemic has shown the need for effective treatments for SARS-CoV-2. RNAi, a natural process that silences gene expression, has great potential for antiviral research and development. siRNAs, synthetic molecules that target specific genes for silencing, have been explored as a promising strategy to combat viral infections. siRNAs have the advantage of being able to target almost any viral gene and being faster to develop than other types of drugs.

However, siRNA-based antiviral RNAi faces several challenges that need to be overcome. A major challenge in antiviral therapy is the continuous mutation of viral genes, which compromises treatment efficacy and narrows therapeutic options. This challenge means the adaptation of viruses to the presence of siRNAs and their evasion of the host cell’s RNA silencing machinery. This is known as the “Red Queen hypothesis,” which describes a molecular “arms race” between viruses and their hosts. Therefore, the development and efficacy of siRNA-based antiviral therapies depend on how well they can prevent viral escape mutants. To cope with the ever-changing viral genome, it is important to target therapeutics that can block viral replication at conserved regions of the genome. Using multiple siRNAs that target different regions can help prevent resistant mutants and increase the chance of maintaining efficacy against new viral strains. Moreover, the efficacy of siRNA-based antiviral therapeutics is one of the important factors in the development of antiviral-resistant escape variants.

The biotechnology industry has invested a lot in developing siRNA-based therapeutic approaches for various diseases, including viral infections, and many studies have focused on antiviral siRNA in recent years. Ongoing research into novel target discovery and siRNA optimization is essential for reducing viral escape potential and achieving durable antiviral efficacy. Despite the challenges of using siRNA as antiviral therapy, the future of RNAi therapeutics looks promising with interdisciplinary collaboration and technological advancements. The use of exogenous antiviral siRNAs to silence genes through the natural RNAi pathway has become a popular technique with great therapeutic potential that may soon be realized. Therefore, it is reasonable to expect that antiviral siRNA applications will have a huge impact on the treatment of viral infections in the future.

Looking forward, it will be crucial to integrate computational prediction, structural modeling, and high-throughput screening to proactively design siRNA combinations that can remain effective against emerging escape variants. In parallel, more research is needed to evaluate these strategies in vivo and ensure their clinical viability across different virus families. Although this review focuses on siRNA-based antiviral approaches, alternative gene-editing technologies such as CRISPR-Cas systems have also shown promise in targeting viral genomes. While CRISPR-based strategies offer certain advantages, such as permanent gene disruption, they also raise unique challenges in terms of delivery and specificity. Future comparative studies evaluating these platforms in the context of rapidly evolving viruses would be valuable.

## Figures and Tables

**Table 1:** Small interfering RNA (siRNA)-based RNA interference strategies for combating viral infections and preventing antiviral resistant escape mutants.

Strategy	Consideration and Options	Expected Effects
Multiple siRNAs	Targeting multiple loci of the same gene [62] Simultaneous targeting several viral genes [63,64,65,66,67] Blocking multiple steps in the viral life cycle [68,69,70]	Synergistic and enhanced inhibition of viral replication [34,67,71,72,73,74,75] Prevent or delay the emergence of escape variants to antiviral siRNAs [37] Significant reduction in the likelihood of a fully resistant mutant emerging [37,76,77]
Targeting highly conserved regions in viral genome	Designing siRNAs for functionally essential and highly conserved regions [78] Using a bioinformatics approach to predict potential mutations [61,79,80] Genes encoding variable regions should be excluded [24,81,82,83] 5′ untranslated region (UTR) and the 3′ UTR [33,36,84,85,86,87,88]	Decrease the possibility of viral escape caused by viral evolution [81,82,83,85] Reduce the number of siRNAs required and consequently lowering the cost [89] A potential of one-for-all therapy for rapidly evolving viruses (“pan-viral” siRNAs) [90,91]
Antiviral resistance screening and rapid identification of new targets	Identify the emergence of escape variants [37,41,77,92,93,94] Screening a library of siRNA sequences against a panel of viral strains, encompassing both wild-type and mutated strains [61,95]	Rapidly adjust if mutations occur in the viral target RNA sequences [95] Development of pinpoint siRNAs that can effectively target multiple viral strains [61] Provide many new targets against constantly evolving viruses [61,95]
In silico (computational) design for optimal siRNA candidates	Data-based computational algorithm supporting the pre-design rooted in bioinformatics, such as stochastic models, viral siRNA databases, siRNA analysis tools, prediction algorithms, and web-based software for antiviral siRNA design developed in order to craft optimal antiviral siRNAs [76,95,96,97,98,99,100,101,102,103,104] Considerations: variations in target RNA, thermodynamics, accessibility of the siRNA and target RNA, and potential off-target effects [95,105,106]	A more cost-effective approach employing statistical bioinformatics analysis of the viral genotype, specifically focusing on the dominant viral genetic sequences present in isolates [107] Use of software for rational design aids in the filtration and selection of potential siRNAs with high accuracy and strength [76,95,96,97,98,99,100,101,102,103,104]
Combination therapy	siRNA-based therapy with other antiviral agents, such as small molecule inhibitors or neutralizing antibodies [74,108,109] siRNAs combined with other antiviral approaches that target critical steps in the viral life cycle [68,69,70]	Reduce the emergence of viral variants that have acquired mutations that render them resistant to siRNA-mediated targeting [68,69,70,74] Enable the use of lower doses of antiviral drugs, thereby reducing the risk of drug-induced side effects [68,69,70] Efficacy of siRNA-based RNAi therapy can be further enhanced [74,108]
Modified siRNA design	Chemical modifications to the siRNA backbone [3,24,33,110,111,112] Conjugates: nanoparticles, aptamers, peptides (receptor ligand), cholesterol, polyethylene glycol, and immune proteins [113,114] Addition of a ‘sticky’ sequence to a chemically synthesized aptamer [112] Unlocked nucleic acid modification [115] Modulating seed-pairing through thermally destabilizing chemical modification [115]	Enhance stability, cellular uptake, and target specificity of siRNAs [115,116,117] Increase the efficacy of the treatment [65,116] Reduce the risk of viral escape variants as well as its toxicity and immunogenicity [112] Improve the pharmacokinetic properties and cellular uptake of siRNAs [24,33,110,111] Facilitate selective siRNA delivery into the target tissue [113] Adapt to the cellular environment and to avoid endosomal sequestration [113] Enhance RISC’s recognition of siRNA, thereby mitigating off-target effects [115,116]
Delivery optimization	Nanoparticles as carriers (nanocarrier systems) such as vesicle-based nanoparticle suspensions post-encapsulation [114,118] Organic origin nanocarrier systems such as lipid-based nanoparticles [72,119,120,121] Inorganic nanoparticles: magnetic nanoparticles, gold nanoparticles, carbon-based nanoparticles, ceramic nanoparticles, metal nanoparticles, and semiconductor nanoparticles [114,122,123]	Deliver siRNAs into targeted sites, enabling precise and focused therapeutic interventions [124,125,126,127] Improve the stability of siRNAs contained therein [128] Combining the targeting properties of nanoparticles with the specificity of RNAi: better therapeutic efficacy with lower side effect profiles [114]
